# Impacts of Intensified Agriculture Developments on Marsh Wetlands

**DOI:** 10.1155/2013/409439

**Published:** 2013-08-20

**Authors:** Zhaoqing Luan, Demin Zhou

**Affiliations:** ^1^Key Laboratory of Wetland Ecology and Environment, Northeast Institute of Geography and Agricultural Ecology, Chinese Academy of Sciences, 4888 Shengbei Street, Changchun 130102, China; ^2^College of Resources, Environment and Tourism, Capital Normal University, Beijing 100048, China

## Abstract

A spatiotemporal analysis on the changes in the marsh landscape in the Honghe National Nature Reserve, a Ramsar reserve, and the surrounding farms in the core area of the Sanjiang Plain during the past 30 years was conducted by integrating field survey work with remote sensing techniques. The results indicated that intensified agricultural development had transformed a unique natural marsh landscape into an agricultural landscape during the past 30 years. Ninety percent of the natural marsh wetlands have been lost, and the areas of the other natural landscapes have decreased very rapidly. Most dry farmland had been replaced by paddy fields during the progressive change of the natural landscape to a farm landscape. Attempts of current Chinese institutions in preserving natural wetlands have achieved limited success. Few marsh wetlands have remained healthy, even after the establishment of the nature reserve. Their ecological qualities have been declining in response to the increasing threats to the remaining wetland habitats. Irrigation projects play a key role in such threats. Therefore, the sustainability of the natural wetland ecosystems is being threatened by increased regional agricultural development which reduced the number of wetland ecotypes and damaged the ecological quality.

## 1. Introduction

Natural ecosystems, especially freshwater ecosystems in the inland flood plain, are undergoing profound and extensive disturbances by humans worldwide [1–5]. A key indicator of these disturbances is that humans extensively reclaim natural wetlands to expand their economic benefits. Therefore, most habitats of natural ecosystems have been changed into farms or urban areas rapidly and continuously [6–8]. The disturbances have been representatively observed in China, the largest developing country in the world. A good example is the shrinking process of the marsh wetland landscapes on the Sanjiang Plain in Northeast China [9, 10].

With its rapid development, China can be regarded as a typical country of most other developing countries in the world. China has experienced high-speed development in the past 30 years. Scientifically assessing or even imagining the impact of urbanization and agricultural reclamation on natural ecosystems is difficult because few countries have comparably rapid and extensive development [11, 12]. During the past 30 years, a large number of natural habitats in China have been reclaimed into cropland, and numerous farmlands have been occupied and then urbanized into towns or cities [7]. With this progress, the Chinese population has rapidly increased and is currently 1.3 billion. The most natural habitats of the wetland ecosystems have been encroached upon during this progress [13]. Though food security is always the top priority for the massive Chinese population [10], the continuous reclamation of the few remaining natural habitats has difficulty meeting the demand of grain production.

Some developing countries, such as China, have published various administrative policies for natural resource protection during their rapid developmental stages. Many natural reserves have been established in the past few years. China has listed the most natural reserves in the world [14]. However, the institutional efficacy of these reserves remains questionable from a scientific perspective [15, 16]. In this paper, the Honghe National Nature Reserve (HNNR) was included within our study area as a wetland reserve. It is also an international wetland listed by the Ramsar Convention. The institutional efficacy of this Chinese natural reserve was the topic of the present study. Researchers analyzed the spatiotemporal changes of the inner and outer landscapes of the reserve and reached some interesting scientific conclusions.

Chinese scholars have recently become concerned about the great changes in the natural marsh wetlands in China. Many papers have reported research results in this field [9, 13, 17–30]. In these studies, some researchers [9, 13, 31–33] analyzed the marsh landscape on the Sanjiang Plain over periods of 20 or even 50 years. Most research approaches were based on theories of landscape ecology. The integration of remote sensing techniques and geographical information systems was applied for the spatiotemporal analysis of marsh landscape segments. Landscape investigators obtain dynamic information on marsh landscapes with the support of remote sensing techniques [34]. However, these studies lack an analysis on the profound driving forces that impact the wetlands and especially lack a correlational analysis of the linkage between policy issues and regional characteristics that deal with the spatiotemporal dynamics of the marsh wetlands. These previous studies focused more on obtaining data and analyzing dynamic wetland landscapes on large regional scales (e.g., 10000 km^2^), which is suitable for the application of remote sensing techniques [35]. Liu and Ma descriptively studied the changes in the natural environments on the entire Sanjiang Plain and its regional ecological response to such changes [9]. Rich survey data and historical statistics of wetlands were used in their study, but the spatiotemporal dynamics of the wetland landscapes were poorly assessed.

Many papers have studied the issue of land use and cover change caused by regional and international urbanization in the past few decades. An abundance of literature has addressed the impact of urbanization and regional development that have encroached on cropland or the reclamation of wild fields in China [10, 11, 36]. Most studies have focused on the spatiotemporal characteristics of changing land use or land cover or have analyzed the relative driving forces. Ecological impact issues related to agricultural activity have long been neglected [14]. Little research has focused on the impact on wetland ecology, linked the dynamics of the marsh landscape over the long term, and studied the driving forces of regional agriculture with a background analysis of historical national policies [7]. This paper provides a case study of the Sanjiang Plain in Northeast China and demonstrates the shrinking process of the typical marsh wetland and other natural landscapes driven by agricultural activity. The ecological impacts on the wetland ecosystems were also analyzed from a regional development perspective. This research will help better understand the gradual evolution of the disturbed natural ecosystems and elucidate the dependence of these natural ecosystems in developing countries. The goal is to help resource administrators determine the evolutionary direction of these ecosystems in the future [37, 38]. An identification of the common characteristics of these natural ecosystems will significantly impact decision making in the management of surviving natural ecosystems in developing countries [38, 39].

The present study sought to achieve three objectives: (1) present the spatiotemporal process of the encroachment of expanding farmland on wild marsh landscapes in the core area on the Sanjiang Plain since 1975, which is a microcosm of shrinking natural wetland ecosystems worldwide; (2) analyze the characteristics of the driving forces that continuously reduce the marsh wetland area in this region, with an emphasis on discussing Chinese policies related to intensified agricultural development on a local scale; and (3) study the negative impact of marsh reclamation on natural ecosystems. An international wetland is used as a typical example to show readers the ecological impact of agricultural activity on marsh wetlands and assess the functional efficacy of this natural reserve.

## 2. Materials and Methods

### 2.1. Study Area

**Figure 1:**
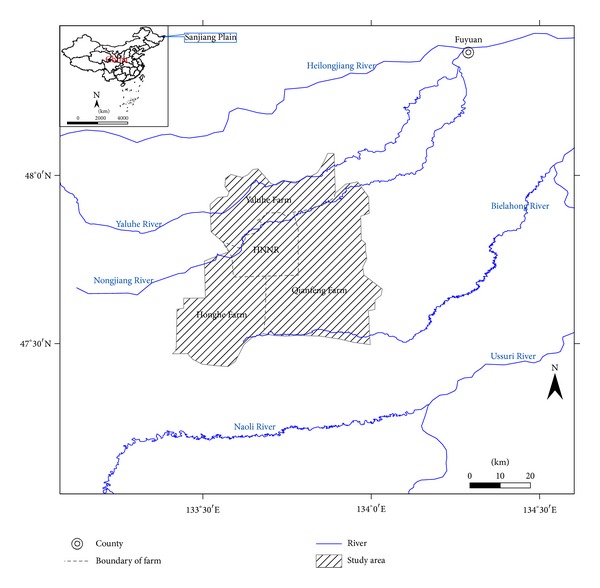
Location of the study area.

The HNNR and its three surrounding farms (Yaluhe Farm, Honghe Farm, and Qianfeng Farm) were selected as our study area. The study area is located in the northeast region of Heilongjiang (47°25′N-48°1′N, 133°18′E-134°5′E), the core area of the Sanjiang Plain (Figure 1). It covers 2416.8 km^2^ in the neighboring area of Tongjiang County and Fuyuan County. This area was a unique marsh wetland landscape 30 years ago. The establishment of local farms coincided with a gradual loss of the marsh wetlands. The establishment of the HNNR was useful for obtaining data on the later progression [40]. Therefore, our study area selection of both the HNNR and its surrounding farms was helpful for comparing and analyzing marsh wetland loss and the negative impacts of neighboring agricultural activity on the marsh landscape in the HNNR.

### 2.2. Methods

The database for this research derived mostly from LANDSAT satellite images. It included one MSS image from July 25, 1975, and two TM images from June 12, 1989, and August 30, 2006. Additional materials used for this research included a geographical map (1 : 100000 scale) and a QuickBird image with a high spatial resolution of 0.61 m from May 16, 2004. All of the landscape maps in raster format that were interpreted from the images were inputted into the ArcGIS 9.2 platform, in which a spatial resolution of less than 0.5 pixels was attained with the aid of a 1 : 10000 scale geographical map. The statistical analysis was complemented with the dynamics of local landscapes during the past 30 years using Excel 2003 software after careful topological examination in the ArcGIS. Data sources about current wetland plant survey and water fowl survey came from our field survey, and the comparable historic data source came from previous research publication (see details in Section 3.4). 

 A classification system of the landscapes needs to be based on the specific objectives of the research, and the hierarchical characteristics of a classification system need to match the corresponding spatial scale of the research. This research focused on the historical exchange between the natural landscapes and artificial landscapes according to the spatiotemporal information generated from the satellite images on three different dates. The landscapes were classified into seven basic classifications that included three ecotypes to analyze the various landscape information in the images. The seven landscape classifications included marsh, river pond, meadow, forest, paddy field, dry farmland, and others. Among these, the river pond classification comprised natural rivers, ponds, and all other artificial water bodies. Few areas included residences in the study area between 1975 and 1989, although this increased in 2006. For an easier historical comparison of the different landscape classifications, residential areas, road areas, and other types of small landscapes were merged into one landscape classification termed “other.”

**Figure 2:**
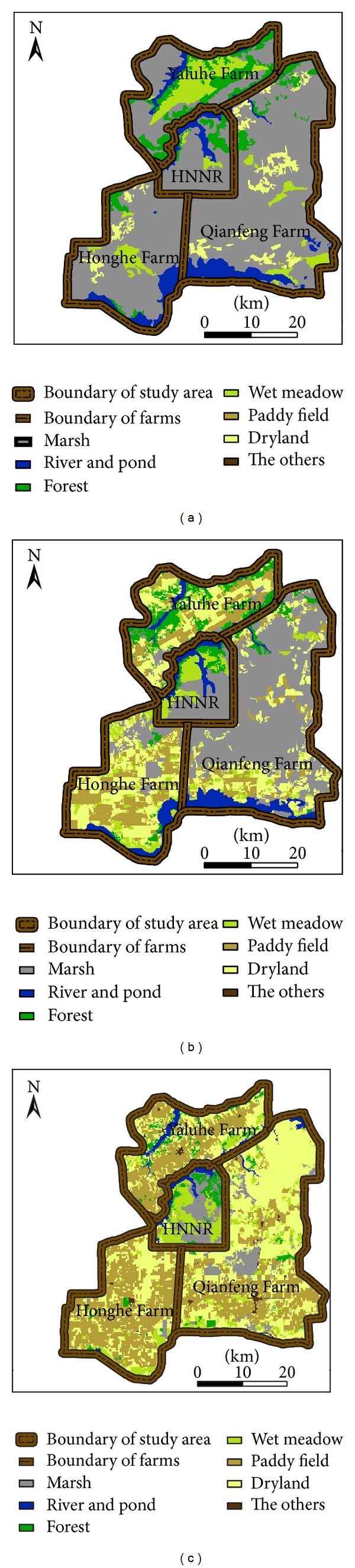
Changes of the wetland landscape within the past 30 years.

The data processing method for this research included constructing a new multiple-band file for georeferenced remote sensing images and a mask for the boundary of the study area within the ENVI 4.0 platform. The mask was applied to the imagery data for the purpose of creating an image-based region of interest in the three specific dates. We utilized the layer stacking tool to construct a new file and then performed rapid filter enhancement on the images to meet the needs of image interpretation. The interpretation signs were then established, based on the images according to different colors, shapes, textures, and field investigation photographs. Manual interpretation was used to obtain the classification maps in raster format to describe the regional wetland landscapes in 1975, 1989, and 2006. The QuickBird image was used for reducing the uncertainty while manually delineating the similar landscapes, such as marsh and meadow. After resetting the digital boundaries of four inner units as the HNNR and three farms within the study area, the three thematic maps of the wetland landscapes were reproduced for dynamic analysis purposes (Figures 2(a), 2(b), and 2(c)). An accuracy estimation was made based on the confusion matrices generated from the database of ground truth and a variety of relevant maps (e.g., the previous land-use maps and a previous classification map of the wetlands) [16, 41]. The results of the accuracy assessment showed that the total classification accuracies reached 92.33%, 92.60%, and 90.41% in 1975, 1989, and 2006, respectively. The kappa coefficients (*N* = 365) were 86.66%, 89.47%, and 86.93%, respectively. Finally, a statistical analysis was performed to present the temporal and spatial changes of the regional dynamic landscapes using Excel 2003 software [41].

## 3. Results and Discussion

### 3.1. Basic Changes of the Landscapes in the Study Area

The progression of gradual marsh landscape loss could be described quantitatively in the study area by comparing and analyzing the dynamic information from the three landscape maps in 1975, 1989, and 2006 (Figures 2(a), 2(b), and 2(c)). The basic marsh landscape in purple changed into farm landscapes in yellow as the present basic landscapes. A very substantial change of the landscapes occurred in the study area, from the 67.1% of the marsh wetland area in 1975 to 73.1% farmland area in 2006. In 1989, the typical marsh wetland loss was 47.4% compared with 1975, and the loss was 89.8% in 2006. The marsh landscape shrank in the HNNR, with a few odd marsh wetlands in the farm areas.

In the past 30 years, a large loss of rivers and ponds occurred during the progression of marsh loss. The landscape in blue lost 53%, and the natural forest loss was 58.2% since 1975. During the progression of the basic natural landscape of the marsh wetlands changing into an agricultural landscape, the dry farmland landscape changed to an increasing number of paddy fields. No paddy fields existed in the study area in 1975, but this landscape comprised one-third of the study area in 2006. A large amount of dry farmland was replaced by paddy fields with the extensive development of agricultural irrigation, which had a very negative impact on the regional marsh wetlands. The few remaining marsh wetlands degraded into meadows because of the loss of healthy habitats attributable to irrigation activity. Therefore, the area of the meadow landscape has seen a nearly 32.3% increase even after most of the original meadows were reclaimed into croplands in the past 30 years.

### 3.2. Progression and Characteristics of Encroachment on Marsh Wetlands

Two matrices of the landscape changes were made for the two periods according to the three landscape maps in 1975, 1989, and 2006 based on interpretations of the satellite images (Table 1, Table 2). From these, we analyzed how the marsh wetlands shrunk while the farm landscapes increased in the study area.

Table 1 shows the apparent loss of marsh wetlands from 1975 to 1989, during which a large amount of marsh wetlands were reclaimed into dry farmland or paddy fields. A 47% loss of the marsh area occurred, and the area of dry farmland increased by 380%, a four-fold increase compared with 1975. In 1989 paddy fields comprised 15% of the study area, while in 1975 almost no paddy fields existed. Twenty percent of the forest area was reclaimed into crop land or for other purposes. The originally existing marsh wetlands were the basic landscape in the study area in 1975, and natural marsh, river, and pond landscapes comprised nearly 90% of the area. Few dry farmlands existed during that time. However, the basic marsh landscape was replaced by a landscape pattern consisting of nearly 40% farmlands in 1989, with dry farmlands being the principle landscape. No significant changes occurred to the other landscapes during this period.

Table 2 shows that the marsh wetlands continued to be lost with a change ratio of over 80%, and the area decreased from 35.3% in 1989 to 6.9% in 2006. At the same time, the other natural landscapes, such as river, pond, and forest, also continuously decreased, with an average loss ratio of 50%. The progression of shrinking natural landscapes coincided with the expansion of farmlands, similar to what happened during the previous period, but some new trends appeared in the change of the landscapes from 1989 to 2006. A substantial change in the farm pattern was a 131% increase in the paddy fields during that period. The dual progression occurred as natural landscapes changed to farm landscapes while dry farmlands were replaced by paddy fields.

### 3.3. Impacts on the Marsh Wetland Habitat due to the Intensified Agriculture Development

**Figure 3:**
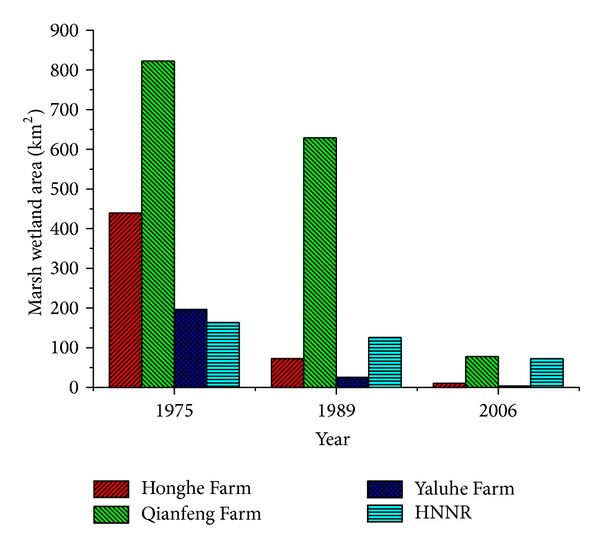
Loss of the marsh wetland of 4 units within the study area in the past 30 years.

The uniformity of the changing landscapes in the study area includes the three surrounding farms that experienced a rapid change from a basic marsh landscape to an agricultural landscape, although they experienced different agricultural progressions and retain different landscape structures as a result of regional development (Figure 3). We concluded that the impacts on the natural wetland habitat caused by marsh reclamation have two characteristics. First, it reduced the area of the marsh wetland habitat directly. Wetland habitats for wildlife and plants were lost largely because of the rapid decrease in the marsh wetlands in the study area. The remaining marsh wetlands became fragmented from a landscape perspective. Second, reclamation weakened the ecological function of the remaining marsh wetlands as habitats. The remaining marsh wetlands lost their healthy habitats because environmental flow was cut or reduced as a result of agricultural irrigation systems that were strengthened continuously on the neighboring farms.

Well known as a natural “gene bank” of most wildlife on the Sanjiang Plain in China, the HNNR was established in 1984 and was upgraded to a national reserve in 1996. In 2002, it was listed in the Ramsar Convention as an international wetland reserve [42]. This reserve is a location of the original typical marsh wetland on the Sanjiang Plain. Compared with the other three farms that have experienced extensive disturbances, the HNNR maintains a basic marsh landscape with less human disturbance. However, its marsh area decreased since the 1980s. Rapidly developing irrigation projects in the surrounding farms cut the water sources to the marsh ecosystem in the HNNR. Therefore, 30% of the marsh wetlands in the HNNR degraded into meadow wetlands [43]. From this research, we can conclude that the establishment of this natural reserve protected the remaining marsh wetlands with the intensified regional agricultural development. Because of the limited reserve area of the HNNR, however, our further analysis showed that marsh wetland ecosystems in the HNNR have been indirectly influenced by the agricultural activity of the surrounding farms that have changed the landscape pattern of the HNNR.

### 3.4. Damage to the Natural Wetland Ecosystem Caused by Local Agricultural Development

Wetlands are well-known habitats of water fowl. The HNNR is a transfer location in East Asia for rare water fowl, such as *Grus japonica* and *Ciconia boyciana*, which have first-order protection status in China [14]. Over 23 species of *Grus japonica* and 400 species of *Ciconia boyciana* were listed in the study area in the early 1970s [44], but only three species of *Grus japonica* and five species of *Ciconia boyciana* were recorded during an uninterrupted observation period in the study area between 2003 and 2004. This represents a nearly 90% loss since the 1970s [36]. For most water fowl, the increasing farmlands and paddy fields cannot replace their natural habitat. The shrinking natural marsh wetlands have an obvious negative impact on the existence of these water fowl [2, 45].

Damage to the wetland habitat for wildlife and plants has also resulted in the loss of rare plant species. Over 50 wetland plant species are listed as endangered at the national level in the region. Both *Dysophylla yatabeana* and *D. fauriei* are now extinct, although they were very common species 30 years ago. The damage to natural habitats harms wetland plants from both biological and ecological perspectives [43]. *Carex lasiocarpa* is a representative species of the local marsh wetland ecosystem. It was recorded in the 1970s as a robust and large plant with an average height of 73.7 cm. However, its height has decreased to an average of 40.5 cm, 33.2 cm shorter than 30 years earlier, according to a field survey conducted between 2003 and 2004 [36]. Its average biomass decreased from 653 g/m^2^ to 403 g/m^2^ (a 30% decrease) in the past 50 years [46]. With regard to plant composition, the richness of the species of wetland plants has also decreased because of decrease in the quality of the wetland habitats. Currently, there is an average of 6.7 species per square meter, a reduction of one species compared with 30 years ago [47]. The biodiversity of the natural ecosystems has definitely been damaged in the region because of the large amount of marsh wetland degradation into meadows. Our future research will precisely assess the weakness of ecological function due to the agriculture development.

### 3.5. Discussion

Marsh wetlands were widespread on the Sanjiang Plain before the 1980s. The growing season is very short (only 4 months) on the Sanjiang Plain. Most of the area in this region is flooded year round because of the extremely cold and moist climate. The rough natural conditions result in few permanent residents in this region. Therefore, the Sanjiang Plain is well known as “The Big Wild” because of its unique natural marsh landscape [9]. With the increasing Chinese population, the country is seriously challenged by the increasing demand for grain. Grain production is a priority for the Chinese government. Therefore, a series of agricultural policies were made to encourage marsh reclamation and the expansion of farmlands for the purpose of agricultural development [13]. The Sanjiang Plain became the highest priority for reclamation because of its abundance of wild land [10]. Within our study area, the Qianfeng Farm was established in 1969. The Yaluhe Farm was established in 1977, and the Honghe Farm was established in 1980. The purpose of these established farms was to reclaim marsh wetlands. However, encroachment on the marsh wetlands was not excessive because of the lower productivity during that time. Local farmers were not willing to produce more grain because of socialist equalitarianism [48–50], and people were busy engaging in various political movements throughout China during that time.

The initial stage of Chinese reform and open policy occurred from 1978 to 1983. During that time, China implemented successful reform of socialistic economic institutions throughout its widespread countryside. Under the reformation rubric, some local farms on the Sanjiang Plain were selected by the central government for pilot projects of modern agricultural farming. The farmers achieved efficient grain production while continuing to reclaim marsh wetlands under reclamation leadership in Jianshanjiang, although this did not reach a climax of regional marsh reclamation [40, 50]. The progression of encroachment on marsh wetlands accelerated on the Qianfeng Farm because of the widespread policy of organizing family farms encouraged by the parent body after 1985 [50]. Following the Qianfeng Farm, the Yaluhe Farm, which was previously a socialist institution, was divided into many small family farms in 1988, and this policy was followed by the Honghe Farm in 1993 [48]. With the new policy, farmers were actively involved in running their family farms. They made investments in various agricultural equipments to expand their own production capacities. Farming efficiency was improved so much that grain production increased during this period [13, 49] by somehow successfully reclaiming marsh wetlands to expand the farmland owned by the family. Encroachments on marsh wetlands most rapidly occurred on the Sanjiang Plain (Figure 3).

The Government of Heilongjiang province published the Regulation of Wetland Protection in Heilongjiang Province on June 20, 2003. It was the first regional regulation on wetland protection by a local government in China. The regulation declared the prohibition of all activities that encroach on wetlands [36]. However, number 1 document from the central government that encouraged an increase in the income of farmers at the national level was published in 2004. The document suggested subsidizing farmers by reducing their agricultural tax [9]. This policy stimulated the farmers' will to increase grain production. Local farmers attempted to reclaim the marsh wetlands to expand their farmland to maximize grain production, even through various illegal means that were against the Wetland Protection Regulation [16, 36]. The technical means of reclaiming marsh wetlands improved substantially during that period, and the modern agricultural facilities helped farmers reduce the cost of marsh reclamation [43]. Marsh reclamation also took disadvantage of both global warming and regional aridity [51]. The gradual illegal encroachment on the few remaining marsh wetlands has not been suspended in the study area, although the reclamation of marsh wetlands has been ceased on a large scale.

**Figure 4:**
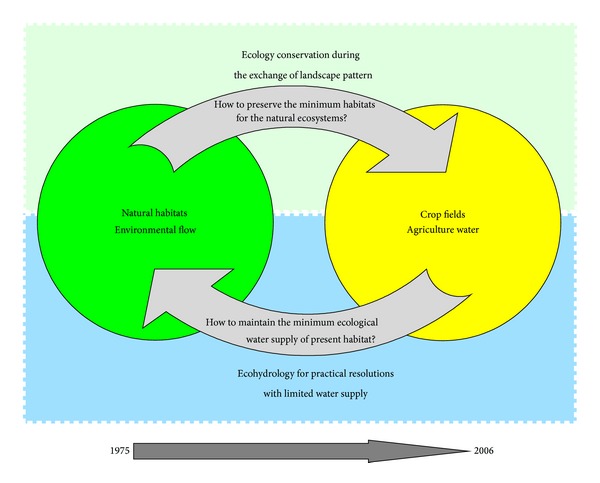
Two key issues in wetland eco-hydrology during the regional process of marsh wetland reclamation.

Marsh reclamation causes obvious negative impacts to wetland ecosystems. Wetlands, the natural habitats of most wildlife and plants, are well known as the “gene bank of wildlife.” Wetlands have significant value for biodiversity in most ecosystems [52–54]. Extensive alterations of both regional hydrology and ecological patterns have occurred at a large scale on the Sanjiang Plain. Marsh reclamation has caused an irreversible and rapid change from a natural ecosystem to an agricultural ecosystem at the regional level. As a consequence of the change, irrigation water has replaced the previous natural environmental flow. However, little research has scientifically assessed the huge disturbance and ecological impact [55, 56]. The challenges include resolving two key scientific issues at the regional level. The first issue is how to preserve a minimum of natural habitats during the rapid progression of ecosystem reductions. The second issue is how to maintain a minimum amount of environmental flow for the remaining natural ecosystems confronted by the increased demand of irrigation water (Figure 4).

## 4. Conclusions

1. Intensified agriculture development has changed a unique natural marsh landscape into an agricultural landscape during the past 30 years in the study area. The reclamation process of marsh wetlands accelerated in response to various national policies that demanded grain production beginning in the 1980s. Ninety percent of the natural marsh wetland area was lost in the study area from 1975 to 2006 while most dry farmland has been replaced by paddy fields.
2. Attempt of current Chinese institution for preserving the regional natural wetlands has achieved limited success. A few wetlands remain healthy because of the establishment of the HNNR, although their ecological quality has declined because of increased threats to the remaining wetland habitats. Irrigation expansion plays a key role in such threats.
3. The sustainability of the natural wetland ecosystems is being threatened by continuous reduction in the wetland habitats number and decline in the ecological quality due to the intensified agriculture development. In the future, it is a big challenge to preserve a minimum of natural habitats during the rapid progression of natural ecosystem reductions while natural resource administrators attempt to maintain a reasonable amount of environmental flow for the remaining natural ecosystems confronted by the increased demand of irrigation water.

## Figures and Tables

**Table 1:** Transformation matrix of landscape and land use within the study area during the period from 1975 to 1989 (unit: km^2^).

1975	1989								
	Marsh	River and pool	Forest	Meadow	Paddy field	Dry farmland	Other types	Total	Proportion (%)
Marsh	759.87	16.18	72.20	137.31	252.9	383.30	0	1621.74	67.10
River and pool	9.20	206.93	7.39	19.29	5.61	7.75	0	256.17	10.60
Forest	24.79	5.45	86.11	14.14	44.27	62.22	0	236.96	9.81
Meadow	36.37	9.95	22.05	72.68	15.74	33.12	0	189.90	7.86
Paddy field	0	0	0	0	0	0	0	0	0
Dry farmland	22.70	0.04	1.85	3.41	32.65	51.38	0	112.03	4.64
Other types	0	0	0	0	0	0	0	0	0

Total	852.92	238.54	189.60	246.82	351.16	537.76	0	2416.80	
Proportion (%)	35.29	9.87	7.85	10.21	14.53	22.25	0	100	

Variation rate (%)	−47.41	−6.88	−19.99	+29.97	/	+380.01	0		

**Table 2:** Transformation matrix of landscape and land use within the study area during the period from 1989 to 2006 (unit: km^2^).

1989	2006								
	Marsh	River and pool	Forest	Meadow	Paddy field	Dry farmland	Other types	Total	Proportion (%)
Marsh	118.68	12.61	23.89	99.06	143.93	453.49	1.26	852.92	35.29
River and pool	22.83	86.06	8.17	45.62	9.96	64.85	1.05	238.54	9.87
Forest	6.05	10.84	39.84	15.12	31.37	86.31	0.08	189.60	7.85
Meadow	13.34	6.74	11.04	61.37	61.14	93.02	0.17	246.82	10.21
Paddy field	0.87	1.06	6.37	14.98	233.82	93.13	0.93	351.16	14.53
Dry farmland	3.76	3.19	9.71	15.13	332.45	162.5	11.02	537.76	22.25
Other types	0	0	0	0	0	0	0	0	0

Total	165.53	120.50	99.02	251.28	812.67	953.30	14.50	2416.80	
Proportion (%)	6.85	4.99	4.10	10.40	33.63	39.44	0.60	100	

Variation rate (%)	−80.59	−49.48	−47.77	+18.07	+131.42	+77.27	/		
